# MicroRNA-34a, Prostate Cancer Stem Cells, and Therapeutic Development

**DOI:** 10.3390/cancers14184538

**Published:** 2022-09-19

**Authors:** Wen (Jess) Li, Xiaozhuo Liu, Emily M. Dougherty, Dean G. Tang

**Affiliations:** 1 Department of Pharmacology and Therapeutics, Roswell Park Comprehensive Cancer Center, Buffalo, NY 14263, USA; 2 Experimental Therapeutics (ET) Graduate Program, Roswell Park Comprehensive Cancer Center and the University at Buffalo, Buffalo, NY 14263, USA; 3 Genetics & Genomics Graduate Program, Roswell Park Comprehensive Cancer Center and the University at Buffalo, Buffalo, NY 14263, USA

**Keywords:** microRNA-34a, microRNA, prostate cancer, cancer stem cells, miRNA therapeutics

## Abstract

**Simple Summary:**

Prostate cancer is still the most common cancer among men in the US. Current standard-of-care therapies for metastatic castration-resistant prostate cancer can offer survival benefits measured only in months. The patients eventually develop drug resistance and tumor relapse. There is strong evidence that during treatment, prostate cancer stem cells may become the predominant population within tumor bulk, and function as the “root cause” for drug resistance. microRNA-34a, a bona fide tumor-suppressive miRNA, represents a potent cancer stem cell suppressor by targeting many molecules essential for cancer stem cell survival. This article will review the tumor suppressive role of miRNA-34a in prostate cancer, and its therapeutic development strategies for advanced prostate cancer patients.

**Abstract:**

Prostate cancer (PCa) is a highly heterogeneous disease and typically presents with multiple distinct cancer foci. Heterogeneity in androgen receptor (AR) expression levels in PCa has been observed for decades, from untreated tumors to castration-resistant prostate cancer (CRPC) to disseminated metastases. Current standard-of-care therapies for metastatic CRPC can only extend life by a few months. Cancer stem cells (CSCs) are defined as a subpopulation of cancer cells that exists in almost all treatment-naive tumors. Additionally, non-CSCs may undergo cellular plasticity to be reprogrammed to prostate cancer stem cells (PCSCs) during spontaneous tumor progression or upon therapeutic treatments. Consequently, PCSCs may become the predominant population in treatment-resistant tumors, and the “root cause” for drug resistance. microRNA-34a (miR-34a) is a bona fide tumor-suppressive miRNA, and its expression is dysregulated in PCa. Importantly, miR-34a functions as a potent CSC suppressor by targeting many molecules essential for CSC survival and functions, which makes it a promising anti-PCSC therapeutic. Here, we conducted a comprehensive literature survey of miR-34a in the context of PCa and especially PCSCs. We provided an updated overview on the mechanisms of miR-34a regulation followed by discussing its tumor suppressive functions in PCa. Finally, based on current advances in miR-34a preclinical studies in PCa, we offered potential delivery strategies for miR-34a-based therapeutics for treating advanced PCa.

## 1. Introduction: PCa Cell Heterogeneity, PCSCs and CRPC

Prostate cancer (PCa) is the second most common cancer in men worldwide and the most commonly diagnosed solid-organ malignancy in men in the US [1]. PCa treatment varies based on pathological parameters, i.e., the Gleason score (GS) grading and staging. Currently, the majority of low-grade (GS6-7) PCa patients are undergoing active surveillance (without any specific treatment), as recommended in clinical guidelines of the American Urological Association (AUA). The majority of these patients show good prognosis without any tumor progression. High-grade tumors (GS9-10) with lymph node (LN) metastasis are treated with drugs named AR signaling inhibitors (ARSIs). In general, ARSIs comprise two subclasses [2]. The first class aims to block AR productions in the testis (e.g., orchiectomy or using LHRH agonists or antagonists) or adrenal gland (e.g., abiraterone acetate), and the former is often called androgen deprivation therapy (ADT). The second class is to prevent the activation of AR signaling through direct binding to AR (e.g., enzalutamide, Enza). Initially, patients with advanced PCa are well responsive to ADT. However, most treated tumors inevitably recur and become resistant to ADT. These patients are subsequently treated with Enza together with chemotherapy (e.g., docetaxel). Castration-resistant PCa, or CRPC, is the general term used to refer to tumors that have failed ADT or ADT/Enza.

For CRPC treatment, ARSIs are still among the standard-of-care therapies. However, therapeutic efficacy is generally short-lived and reported survival benefit is measured only in months. What is attributed to ARSI resistance? One of the under-appreciated mechanisms is related to cell heterogeneity. Cancer cells are inherently heterogeneous both *in vitro* and *in vivo*, exhibiting unique traits phenotypically, epigenetically, and functionally [3]. Therapy resistance is significantly influenced by intra-tumor heterogeneity [4]. Over the course of PCa development and progression, from treatment-naïve primary tumors to CRPC to metastatic CRPC (mCRPC), significant heterogeneity in AR and PSA expression have been demonstrated [2,5,6].

Within tumor heterogeneity are cancer stem cells (CSCs), which generally represent a small subpopulation of the bulk tumor cells in early-stage treatment-naïve tumors. CSCs are operationally defined as the stem-like cancer cells that possess some or most of the normal stem cell properties such as relative quiescence but with great proliferative potential, the ability to self-renew and differentiate, and, importantly, the capability to regenerate and long-term propagate tumors [3]. Our group provided evidence that in untreated prostate tumors the PSA^−/lo^ PCa cell population harbors authentic prostate cancer stem cells (PCSCs) that possess the capability of long-term self-renewal, tumor propagation *in vivo*, and inherent therapy-resistance [7]. Additionally, castration can reprogram PCa cells such that CRPC, which can manifest as either AR^+/hi^ or AR^−/lo^, can be highly enriched in PCSCs. For example, our group reported AR^−/lo^PSA^−/lo^ CRPC models that exhibit prominent upregulation of CSC molecules and are refractory to Enza treatment *de novo* [5]. PCa progression may also be accompanied by increased ‘stemness’ based on transcriptome-based stemness score analysis, which might reflect increased CSC abundance in advanced and treatment-failed tumors [8]. These observations reinforce the notion that spontaneous tumor progression and therapeutic treatments may induce plasticity in non-CSCs and reprogram them into PCSCs. Consequently, PCSCs may become the predominant cell population in ARSI resistant tumors, and the “root cause” for drug resistance. However, current ARSI-based treatment regimens primarily target AR^+/hi^ CRPC cells or clones but largely ignore AR^−/lo^ cells that represent more aggressive PCSC subsets.

microRNA-34a (miR-34a) is a potent CSC suppressor by targeting many molecules essential for CSC survival and functions, which makes it a promising anti-PCSC therapeutic. However, there has not been a comprehensive review focusing on miR-34a’s role and potential therapeutic application in the context of PCa, especially with respect to targeting PCSCs. In this review, we provide an updated discussion of the mechanisms of miR-34a regulation followed by elaborating its tumor suppressive functions in the context of PCa and PCSCs. Finally, we discuss current miR-34a preclinical studies in PCa and offer potential delivery strategies for miR-34a based therapeutics for treating advanced PCa.

## 2. miR-34a Expression Decreases with Increasing PCa Grade but Correlates with Better Patient Survival

**Figure 1:**
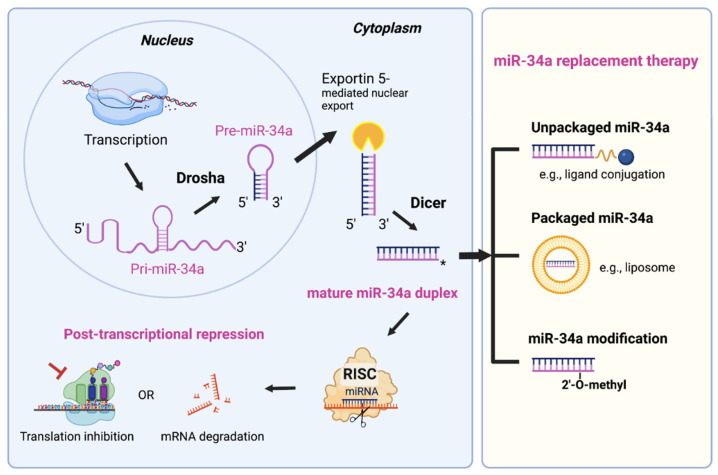
miRNA biogenesis using miR-34a as an example and miR-34a replacement therapy. *miR-34a* encoding gene is transcribed as the primary transcript (pri-miR-34a) in the nucleus. Next, pri-miR-34a (~110 nucleotide) transcripts undergo the first cleavage by the RNase III enzyme Drosha in complex with the dsRNA-binding protein DGCR8 (also known as Pasha). The resulting product is ~70 nucleotide stem-loop-structured pre-miR-34a precursor. Pre-miR-34a is then transported to the cytoplasm by Exportin 5, where they undergo the second and final cleavage, catalyzed by the RNase Dicer. The resulting 22 bp RNA duplex consists of the mature miR-34a (guide strand) and its passenger strand (miR-34a*), which is released and degraded. Mature miR-34a is incorporated into the RNA-induced silencing complex (RISC), which mediates silencing activity of target mRNAs by either inhibiting translation or inducing mRNA degradation. For the miR-34a replacement therapy (right), the delivery platforms include unpackaged (i.e., vehicle-free) delivery and packaged vehicle delivery. In addition, chemical modification of miR-34a oligos is necessary for enhancing the stability.

**Figure 2:**
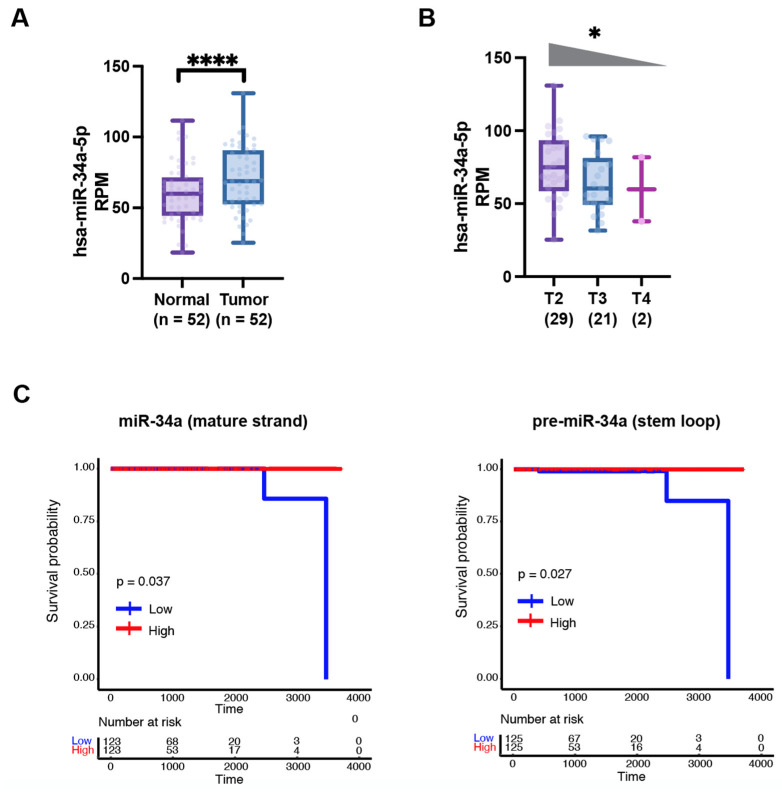
miR-34a expression decreases with increasing tumor grade and correlates with better PCa patient survival. (**A**) Mature miR-34a expression levels are increased in treatment-naive primary tumors compared to matching normal tissues in TCGA (****, *p* < 0.0001, Student’s *t*-test). (**B**) Mature miR-34a expression levels decrease with increasing tumor stage in TCGA (*, *p* < 0.05, Jonckheere–Terpstra trend test). (**C**) Reduced levels of mature miR-34a (left) and pre-miR-34a (right) correlate with worse patient overall survival (TCGA dataset). *p*-Value was determined using the Log-Rank test.

**Figure 3:**
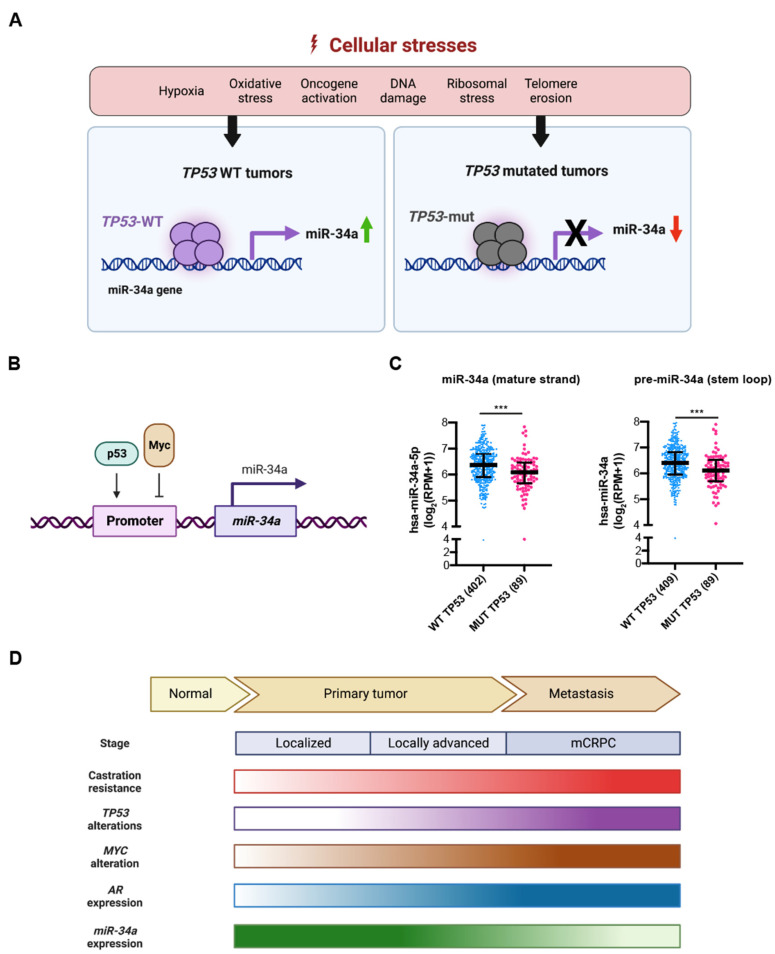
miR-34a may represent an effective ‘replacement’ therapeutic in *TP53* mutated cancers. (**A**) In response to cellular stress signals, p53 is activated and directly binds to the promoter of *miR-34a* gene and induces miR-34a expression. Consequently, miR-34a expression is reduced in *TP53* mutated tumors. (**B**) Schematic illustrating that p53 transcriptionally induces whereas Myc suppresses miR-34a expression by directly binding to the promoter region of the *miR-34a* gene. (**C**) Both mature miR-34a (left) and pre-miR-34a (right) expression levels are decreased in *TP53* mutated prostate tumors compared to *TP53* WT prostate tumors in TCGA (***, *p* < 0.001, Student’s *t*-test). (**D**) PCa progression and castration resistance are accompanied by increasing *TP53* mutations and decreasing miR-34a levels but gradually increased *AR* and *MYC* expression and activity. In principle, miR-34a should be an effective therapeutic for *TP53*-mutated PCa.

MicroRNAs (miRNAs) are ~22-nucleotide (nt) non-protein coding RNAs and critical posttranscriptional regulators of gene expression. The biogenesis of miRNA is a multi-step process that initially consists of generation of pre-miRNA in the nucleus by Drosha complex from the primary miRNA transcript (pri-miRNA) and subsequent Exportin 5-mediated nuclear export of pre-miRNA (Figure 1, left). The mature 20–25 bp miRNA duplex is then produced in the cytoplasm after the final cleavage by the RNase Dicer (Figure 1, left). Mature miRNAs elicit gene silencing function through either translation inhibition or messenger RNAs (mRNAs) degradation. A seed region (nt 2–8) of miRNA can recognize partially complimentary sequences in the 3’-untranslated regions (3’-UTR) of their target mRNAs. miRNAs are dysregulated in almost all malignancies, suggesting their roles in tumorigenesis [9,10,11]. miR-34a, a bona fide tumor-suppressive miRNA, is downregulated in a variety of cancers including hematologic malignancies [12]. We investigated the miR-34a expression pattern and its effect on PCa survival in TCGA (The Cancer Genome Atlas) dataset via bioinformatic approach (Figure 2A–C and Figure 3C). Interestingly and unexpectedly, we found that the expression levels of mature (22 nt) miR-34a (i.e., hsa-miR-34a-5p) are elevated in primary PCa samples compared to matching benign/normal prostatic tissues in TCGA (Figure 2A). This miR-34a expression pattern in treatment-naïve PCa is reminiscent of the expression pattern of LRIG1, an AR-regulated feedback prostate tumor suppressor [13]. PCa development involves activation of multiple oncogenic signaling pathways such as AR and c-Myc, and miR-34a upregulation, like elevated LRIG1 expression, might well represent a feedback inhibitory mechanism to antagonize oncogenic signals driven by MYC and AR (see below). In this sense, the elevated miR-34a in primary prostate tumors would still behave as a tumor suppressor. In support, miR-34a expression is found to negatively correlate with the tumor (T) stage in PCa samples (Figure 2B). To lend further support, both mature miR-34a and the stem-loop pre-miR-34a levels positively correlate with PCa patients’ overall survival (Figure 2C), suggesting that miR-34a is a prostate tumor suppressor. Extensive research has demonstrated that miR-34a suppresses tumor growth and cancer progression by directly targeting many factors involved in cancer-relevant cellular processes [14]. Notably, miR-34a functions as a potent CSC suppressor via targeting many molecules essential for CSC survival and functions in prostate, colon, breast, lung, and other cancers [14]. The role of miR-34a in inhibiting PCSCs and PCa metastasis was first reported by our group [15], indicating that miR-34a replacement therapy could be a novel therapeutic strategy to target PCSCs and aggressive PCa (Figure 1, right).

## 3. Mechanisms of miR-34a Regulation

miR-34a expression is regulated transcriptionally and epigenetically. At the transcriptional level, miR-34a expression is dictated by transcription factors that bind to its promoter region [14]. In the context of cancer, miR-34a expression is largely regulated through epigenetic mechanisms, involving various long non-coding RNAs (lncRNAs) and histone-modifying enzymes.

### 3.1. Transcriptional Regulation of miR-34a Expression

Expression of miR-34a is predominantly regulated by the tumor suppressor p53, which is activated by a multitude of cellular stresses (Figure 3A). *miR-34a* is a direct transcriptional target of p53, which binds to several canonical p53 binding sites in regions proximal to the *miR-34a* promoter [16,17,18] (Figure 3B). miR-34a induction by DNA damage and oncogenic stresses depends on p53 both *in vitro* and *in vivo* [16]. In addition, there is a positive feedback loop between p53 and miR-34a that is partially contributed by miR-34a-mediated suppression of Mdm4, a negative p53 regulator [19]. *mir-34* deficiency alone did not display significant tumor-promoting effect in a *Kras*-induced mouse lung cancer model. *mir-34* deficiency, on the other hand, enhanced tumorigenesis only when *Trp53* was haplo-insufficient, suggesting that the defective feedback loop between p53 and miR-34a can promote oncogenesis in context dependent manner [19]. *TP53* mutations are enriched in PCa with progression [20,21,22,23,24]. For example, 8% *TP53* mutations were observed in localized PCa, which was increased to 40% in a cohort of 429 metastatic PCa patients [21]. Of significance, we found that the levels of both mature miR-34a and pre-miR-34a correlated well with the *TP53* status in PCa such that their expression levels were significantly reduced in *TP53* mutated tumors (Figure 3C). In comparison to LNCaP cells that express wild-type *TP53*, miR-34a expression is also decreased in *TP53*-null PC-3 cells and *TP53*-mutated DU145 cells [15,25,26]. These results suggest a clear reciprocal relationship between *TP53* status and the miR-34a levels during PCa progression (Figure 3D), and support the use of miR-34a replacement therapy in *TP53*-mutated advanced PCa.

Of interest, miR-34a expression remained high in the brain, testis, and lung tissues in *Trp53* deficient mice, suggesting the existence of p53-independent mechanisms that determine basal *miR-34* transcription [27]. C-Myc, the protein encoded by the *MYC* oncogene, is a transcription factor that regulates cell growth, cell cycle, and metabolism. In contrast to p53, Myc represses *miR-34a* transcription by binding to conserved promoter region of *miR-34a* [28] (Figure 3B). Myc-mediated repression of miR-34a was observed in many cancers such as lymphoma [28,29]. *MYC* amplification was found in 46% of advanced PCa samples but only 25% of clinically localized prostate tumors, [21], suggesting that *MYC* amplification accompanies as well as, likely, drives PCa progression. Further studies have confirmed that *MYC* is one of the genes significantly amplified in CRPC versus primary PCa [30], suggesting that repression of miR-34a expression may represent a fundamental component of the *MYC* tumorigenic program (Figure 3D).

**Figure 4:**
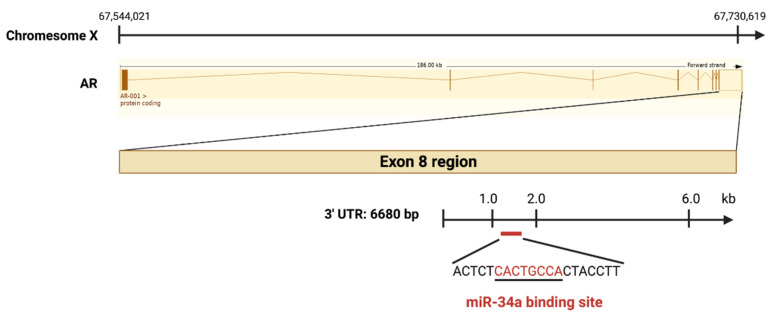
AR is a direct target of miR-34a. The *AR* gene is located on the X chromosome at Xq11–12, and the pre-spliced AR-001 transcript is 186 kb long. The 3’-UTR region (6680 bp), located downstream of Exon 8, harbors a miR-34a binding site (marked in red).

AR, a steroid hormone receptor normally activated by androgens, is critical for PCa development, progression, and therapy response [5]. In normal prostate, androgens promote survival and differentiation, but during PCa development, AR drives uncontrolled cell growth. miR-34a was reported to mediate AR-dependent, p53-induced apoptosis in PCa cells [5]. In the study, DNA double-strand break inducing agent doxorubicin (DOX) leads to p53-induced apoptosis by upregulating miR-34a. However, no increase of miR-34a was found in PCa cells with AR knocked down after DOX treatment. Importantly, DOX did not induce miR-34a expression in LNCaP cells grown in androgen-free medium or in AR-negative PC-3 and DU145 cells [31], which implies that AR might positively regulate miR-34a expression. In contrast, another study reported an AR-miR-204-XRN1-miR-34a feedback loop in neuroendocrine-like PCa, showing negative correlation between AR and miR-34a [32]. In this loop, AR represses miR-34a by upregulating XRN1, while XRN1 raises AR expression by reducing expression of miR-34a [32]. It appears that AR might regulate miR-34a in a context dependent fashion. On the other hand, AR is proven to be a direct target of miR-34a, which binds to the 3’-UTR of *AR* exon 8 region [33] (Figure 4). The mutual regulation between AR and miR-34a implies that reduced miR-34a may further contribute to increased AR expression and activity during PCa progression (Figure 3D).

miR-34a expression has also been reported to be transcriptionally repressed by STAT3, which is involved in IL-6-triggered IL-6R/STAT3/miR-34a feedback loop that promotes EMT-mediated colorectal cancer invasion and metastasis [34]. This study also showed that deletion of *mir-34a* facilitated tumor invasion in a mouse model of colitis-associated cancer, providing *in vivo* evidence for the tumor-suppressive functions of *mir-34a*. The IL-6R/STAT3/miR-34a feedback loop was also reported in PCa cell lines and to be associated with a mesenchymal phenotype [34].

### 3.2. Epigenetic Regulation of miR-34a Expression

Hermeking and his team were the first to report miR-34a downregulation in some cancers as a result of aberrant CpG methylation of its promoter [26]. CpG methylation in the *miR-34a* promoter and concurrent loss of miR-34a expression was displayed in 19 out of 24 (79.1%) primary PCa [26]. Further study confirmed that miR-34a is epigenetically downregulated by DNA methylation in PCa compared to paired normal tissues [35]. The lncRNA HOTAIR was reported to promote tumor metastasis by regulating EMT-related genes as well as *miR-34a*. HOTAIR recruits and binds to polycomb repressive complex 2 (PRC2), which represses miR-34a by enhancing DNA methylation of the *miR-34a* promoter [14]. Another lncRNA, Lnc34a, can epigenetically silence miR-34a expression via recruiting DNMT3A through PHB2 and HDAC1 to methylate and deacetylate the *miR-34a* promoter simultaneously [36].

5-aza-2’deoxycytidine (5Aza-2’dC) is a DNA methyltransferase inhibitor that reactivates the expression of genes silenced by CpG methylation. 5Aza-2’dC treatment reversed miR-34a silencing and upregulated miR-34a expression in PC-3 and LAPC4 cells [26]. Likewise, BR-DIM, a novel demethylating agent, increased the expression of miR-34a through demethylation of the *miR-34a* promoter in C4-2B and LNCaP cells, and its efficacy was shown to be more effective than 5Aza-2’dC [37]. Methylation-induced silencing of *miR-34a* promotes chemoresistance by directly upregulating ATG4B-induced autophagy through AMPK/mTOR pathway in PCa cells [35]. Upregulation of miR-34a by 5Aza-2′dC or ectopic miR-34a treatment sensitized PC-3 and DU145 cells to chemotherapy (e.g., DOX). Abnormal estrogen signaling, due to the decrease of androgen in favor of estrogen during aging, results in almost complete silencing of miR-34a in aggressive PCa [38]. Estrogen-dependent repression of miR-34a is prevented by treatment with HDAC inhibitors [38]. These findings suggest that epigenetic drugs as well as miR-34a replacement therapy can be beneficial to PCa patients with epigenetically downregulated miR-34a, regardless of the *TP53* status.

## 4. Tumor Suppressive Role of miR-34a in PCa

Due to aberrant transcriptional regulation, genomic deletions, and/or promoter hypermethylation, as discussed above, miR-34a expression is downregulated in a wide range of human cancers. This implies that *miR-34a* deficiency may have a function role in tumorigenesis. He and her group were the first to report phenotypic characterization of *mir-34*-deficient mice. Interestingly, mouse embryonic fibroblasts lacking *mir-34* did not show any abnormalities in p53-dependent cell cycle arrest, apoptosis, or replicative senescence [39]. Moreover, the *mir-34a^−/−^*; *mir-34b/34c^−/−^* mice did not display increased susceptibility to spontaneous, irradiation-induced, or c-Myc-initiated tumorigenesis [27]. In these contexts, it is conceivable that *mir-34* loss alone is insufficient for tumorigenesis and that the redundant pathways downstream from p53 could compensate for *mir-34* deficiency *in vivo*. Indeed, in a *Kras^G12D^* lung cancer model, *Kras^LSL-G12D/+^*; *Trp53^+/−^*; *mir-34a^−/−^* mice exhibited increased tumor area and the number of high-grade tumors than *Kras^LSL-G12D/+^*; *Trp53^+/−^* mice, suggesting that *mir-34a* deficiency accelerated tumor progression when p53 response is compromised [19]. In addition, loss of *mir-34a* promoted tumor invasion in a mouse model of colitis-associated cancer [34]. Similarly, *mir-34a* deficiency promoted colon tumorigenesis after Citrobacter rodentium infection [40]. These observations suggest that miR-34a is a tumor suppressor.

Cheng et al. were the first to elucidate the role of miR-34a in PCa by using *mir-34a* deficient genetic mouse models [41]. In the study, it was demonstrated that miR-34 suppressed prostate tumorigenesis in cooperation with p53 by controlling the prostate stem cell compartment via MET [41]. Mice with *mir-34* and *Trp53* specifically inactivated in prostate epithelium led to expansion of the prostate stem cell compartment, development of early invasive adenocarcinomas, and high-grade prostatic intraepithelial neoplasia. Interestingly, any atypical lesions were not displayed in mice losing all *mir-34* genes in the prostate epithelium (*mir-34^PE−/−^* mice) by 15 months of age [41], suggesting that *mir-34* deficiency alone is insufficient to drive prostate tumorigenesis. Mice with lacking both *Trp53* and *mir-34* promoted self-renewal, MET-dependent growth, and motility of prostate stem cells in the proximal region of prostatic ducts [41]. These findings provide direct genetic evidence that *mir-34a* is a bona fide tumor suppressor, and demonstrate that miR-34 can repress PCa development by cooperating with p53.

**Figure 5:**
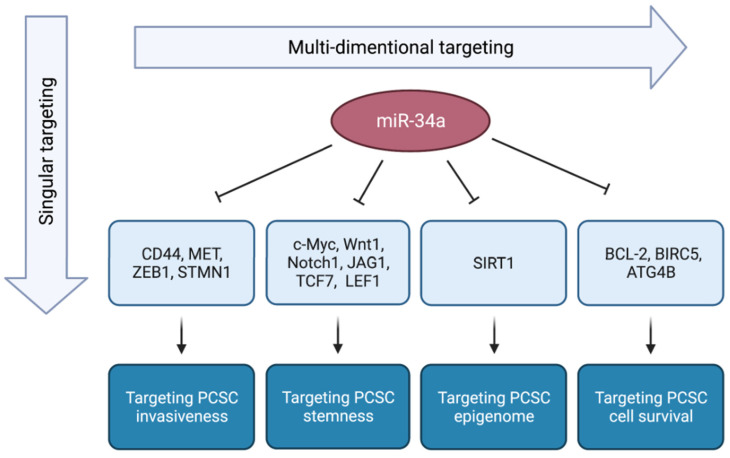
miR-34a potentially offers multidimensional anti-PCSC effects. Current CSC-targeting therapy approaches focus on a single characteristic of CSCs. miR-34a replacement therapy, on the other hand, could provide multifaceted antitumor effects by targeting several cellular processes that are vital for PCSC functions and activities (Adapted from [14]).

miRNA expression profiling in PCSCs was first conducted by our group [15]. Through an unbiased profiling, we revealed that miR-34a is commonly underexpressed in all five PCSC populations purified from PCa xenografts, including three CD44^+^ populations, CD133^+^ cells, and α2β1^+^ population [15,42]. The underexpression of miR-34a was further confirmed in CD44^+^ PCa cells purified from prostate tumors [15]. These findings indicate that miR-34a is devoid in PCSCs within the prostate tumor bulk and that miR-34a reintroduction would elicit antitumor effect by targeting PCSCs. In fact, extensive studies have shown that miR-34a is a potent PCSC suppressor by targeting critical cellular processes essential for CSC survival and functions (Figure 5).

### 4.1. Targeting Invasiveness and Metastasis

Our group was the first to report the role of miR-34a in inhibiting PCSCs and metastasis. Ectopic expression of miR-34a in bulk PCa cells or purified CD44^+^ cells exerted significant antitumor effects on tumor growth and metastasis *in vivo* [15]. In contrast, employing antagomirs in bulk or CD44^−^ PCa cells to neutralize endogenous miR-34a boosted tumor regeneration and metastasis. Strikingly, systemic delivery of miR-34a through tail vein repressed lung metastasis, thereby prolonging the survival of mice bearing orthotopic human prostate tumors. Mechanistically, miR-34a suppressed PCSC properties by inhibiting prostasphere formation, migration, and invasiveness of CD44^+^ PCa cells, as well as serial tumor transplantation. Notably, CD44 was found to be a direct target of miR-34a [15]. Hence, our findings demonstrate that miR-34a is a PCSC suppressor that inhibits tumor development and metastasis by directly targeting CD44. The work provides compelling evidence for the development of miR-34a as a novel therapeutic against aggressive and metastatic PCa.

MET plays a vital role in promoting cell motility, invasion and metastasis of CSCs [43]. It was reported that PCa-propagating cells express MET, and depletion of MET results in a decrease in prostasphere formation [44]. Previous studies identified MET as a target of p53 and miR-34 [16,45,46]. Cheng et al. revealed that miR-34a cooperates with p53 in suppression of prostate tumorigenesis by targeting MET [41]. Loss of *mir-34a* and *Trp53* promoted MET-dependent growth, self-renewal, and motility of prostate stem/progenitor cells, leading to tumor development [41]. In addition, ectopic overexpression of miR-34a decreased MET mRNA level in PC-3 and PC-3MM2 cells [47], whereas MET overexpression reversed miR-34a-induced suppression of invasion in PC-3 cells [48]. Importantly, systemic introduction of miR-34a decreased PCa bone metastasis by suppressing MET [47].

Epithelial-to-mesenchymal transition (EMT) is a unique process of cellular reprogramming and phenotypic changes accompanied by the loss of epithelial markers, leading to an enhancement in tumor invasive and metastatic ability. ZEB1, one factor of the ZEB family, plays a role in accelerating cell migration and invasion by promoting EMT. Zhang et al. reported that miR-34a enhanced docetaxel sensitivity in docetaxel-resistant PCa cells both *in vitro* and *in vivo* through inhibiting EMT by targeting ZEB1 [49].

Oncoprotein STMN1 (also known as stathmin 1 and oncoprotein 18) plays a role in cell proliferation, motility, and cancer metastasis [50]. STMN1 expression is elevated in metastatic PCa, and knockdown of STMN1 resulted in reduced proliferation and invasion of PCa cells *in vitro* as well as tumor growth and metastasis *in vivo* [50]. miR-34a reduced cell proliferation and colony formation in DU145 and PC-3 cells by directly targeting STMN1 [50], suggesting its negative regulation of invasion and metastasis through STMN1.

### 4.2. Targeting Stemness

Cancer ‘stemness’ is defined as CSC traits and properties in cancer regulated by stem cell factors, and many studies have reported that miR-34a represses cancer stemness by targeting the stem cell factors. Myc is highly expressed in PCa cells with stem-like and tumor-initiating properties [51]. Myc silencing using a promoter-targeting siRNA reduced the fraction of PCSCs, leading to reduced self-renewal, tumor-initiating and metastatic capabilities, indicating a causal role of Myc in PCSC maintenance [51]. Yamamura et al. reported that miR-34a inhibited invasion by suppressing RhoA expression through directly targeting c-Myc [48]. miR-34a suppressed the recruitment of the c-Myc–Skp2–Miz1 complex to the *RhoA* promoter, resulting in downregulation of RhoA [48]. In addition, delivery of miR-34a reduced subcutaneous PC-3MM2 prostate tumor burden by inducing apoptosis and downregulating c-Myc [47].

In cancers, CSCs often exhibit dysregulation in critical developmental and signaling pathways including WNT and NOTCH [14]. WNT signaling is important for stemness maintenance, and aberrant WNT signaling is driven by stabilized β-catenin which in turn mediates inappropriate gene activation. Accumulated cytoplasmic β-catenin translocates into the nucleus where it associates with members of the T-cell factor (TCF) and lymphoid enhancer factor (LEF). Subsequently, the β-catenin-TCF/LEF complex activates the transcription of target genes including c-Myc and cyclin D1. miR-34a overexpression induced G2 cell cycle arrest and promoted apoptosis in PC-3 cells by inhibiting Wnt/β-catenin activity [52]. Mechanistically, miR-34a directly targeted WNT1 and inhibited translocation of β-catenin into the nucleus, leading to suppression of PC-3 cell proliferation and migration [52]. Furthermore, TCF7, a canonical WNT response gene and a mediator of bone metastasis, has been reported to be a direct target of miR-34a [53]. Downregulated miR-34a is associated with activated WNT signaling in metastatic PCa samples compared to primary tumors and normal prostatic tissues [53]. Ectopic miR-34a expression inhibited bone metastasis and reduced cancer cell proliferation in a Ras-dependent xenograft model by repressing WNT/TCF7 signaling [53]. LEF1, another key transcription factor in the WNT signaling pathway, has been reported to regulate cancer invasion and metastasis [54]. miR-34a suppressed EMT, migration and invasion in PC-3 cells by targeting LEF1 [55].

NOTCH signaling is activated during PCa development and progression. Knockdown of NOTCH1 in C4-2R cells improved Enza sensitivity by decreasing cell proliferation and promoting apoptosis, suggesting that NOTCH1 signaling may contribute to drug resistance in PCa [56]. Ectopic miR-34a expression reduced stemness (colony formation) and enhanced sensitivity to paclitaxel by directly targeting NOTCH1 and JAG1 both *in vitro* and *in vivo* [57], indicating that miR-34a may overcome drug resistance and improve therapeutic efficacy for CRPC.

### 4.3. Targeting Epigenome

Sirtuin-1 (SIRT1), a class-III histone deacetylase, has a key role in the epigenetic regulation of tissue homeostasis and many diseases by deacetylating both histone and non-histone substrates [58]. SIRT1 overexpression sustains CSC activities in different cancers [59]. SIRT1 is reported to be upregulated in PCa [58]. miR-34a overexpression induced cell-cycle arrest and growth inhibition and attenuated chemoresistance to anticancer drug camptothecin by targeting SIRT1 [25]. Intriguingly, miR-34a-induced SIRT1 inhibition occurred at the transcriptional rather than post-transcriptional level despite the presence of a potential miR-34a binding site within its 3′-UTR [25]. Another study also revealed that miR-34a attenuated paclitaxel resistance in PC-3 cells by targeting SIRT1 [60]. Systemic administration of micelles carrying paclitaxel and miR-34a inducer rubone inhibited orthotopic prostate tumor growth by inhibiting CSC population and downregulating SIRT1, cyclin D1 and E-cadherin [61].

### 4.4. Targeting Cell Survival

Our group previously demonstrated that BCL-2, an anti-apoptotic protein as well as a CSC marker, is exclusively upregulated in CRPC patient, that BCL-2 is directly induced by Enza treatment, and that BCL-2 is upregulated in both Enza-resistant AR^+/hi^ and Enza-insensitive AR^−/lo^ CRPC models [5]. These findings suggest that BCL-2 plays a vital role in drug resistance and cancer progression. Corcoran et al. reported that docetaxel-resistant variants of PCa cell lines harbored lower miR-34a levels as well as elevated expression of BCL-2 compared to the respective nonresistant cell lines, indicating a reciprocal relationship of miR-34a and BCL-2 in PCa chemoresistance [62]. Further study confirmed that miR-34a sensitized PCa cells to apoptosis-inducing drugs such as camptothecin by directly targeting BCL-2 [60,62]. Ectopic expression of miR-34a in paclitaxel-resistant PC-3 cells decreased BCL-2 as well as the RNA binding protein, human antigen R protein (HuR) [60]. Notably, knockdown of HuR via siRNA also reduced BCL-2 mRNA levels [60], indicating that miR-34a may regulate BCL-2 in both a direct and indirect manner.

Another anti-apoptotic protein, BIRC5, is upregulated in metastatic PCa and in prostate tumors with higher clinical grade [53]. The expression levels of BIRC5 were inversely correlated to miR-34a expression in metastatic PCa patients [53]. BIRC5 was previously reported to be a target gene of miR-34a in breast and colorectal cancers, and this relationship was also confirmed in PCa [53]. miR-34a overexpression induced dramatic increase in apoptosis in Ras signaling- activated PCa cells, and the apoptotic effect of miR-34a was reversible by overexpression of BIRC5 [53].

Autophagy, a survival mechanism conserved from yeast to mammals, is known to be widely exploited by cancer cells [63]. The association between cancer cell survival and autophagy can be partly explained by the role of autophagy in protecting cells from undergoing programmed cell death [63]. Liao et al. found that ectopic expression of miR-34a inhibited autophagy by downregulating ATG4B, Beclin-1 and LC3B II/I in DU145 and PC-3 cells [35]. Furthermore, ATG4B is directly targeted by miR-34a [35]. Mechanistical studies showed that overexpression of miR-34a resulted in a significant downregulation of p-AMPK and upregulation of p-mTOR [35], indicating that miR-34a may regulate ATG4B-induced autophagy through the AMPK/mTOR pathway.

## 5. miR-34a Therapeutic Development for Aggressive PCa

### 5.1. miR-34a Replacement Therapy

miR-34a replacement therapy is a new therapeutic concept, which aims to restore a loss of function to inhibit cancer growth by reintroduction of tumor suppressor miR-34a in tumor cells. A miR-34a therapeutic is a mature double-stranded duplex that does not require further processing by Dicer and can directly elicit gene silencing effect by entering the RISC (Figure 1, left). Dicer, an important endoribonuclease required for miRNA biogenesis, is often dysregulated in cancers. Thus, miR-34a replacement therapy could be beneficial to the cancer patients with Dicer dysfunction through circumventing endogenous miRNA processing steps.

The delivery system for miR-34a therapeutic can be categorized into two major platforms (Figure 1, right). One platform is packaged vehicles, including liposomes and other types of nanoparticles. The ability of liposomes to deliver a variety of payloads, including chemotherapy drugs, oligonucleotides, DNA, siRNA, and proteins, has made them the most widely used method for delivery of therapeutic agents [64]. Current delivery systems of miR-34a mainly utilize the packaged vehicle strategy [14]. The other platform is unpackaged or ligand-conjugates, such as folate [65,66]. Despite extensive research in the field, the translation of miR-34a therapeutics into the clinic has been hindered by several issues including delivery vehicle-associated toxicity, inefficient cellular uptake and serum stability, and limited specificity to targeted tumors. The stability problem is due to the sensitivity of unmodified miR-34a oligos to nuclease-mediated degradation, thereby requiring repetitive cycles of high-dose to achieve the desired therapeutic response [66]. Thus, chemical modifications have been pursued to overcome the issue (Figure 1, right). For example, introducing 2’-O-methyl and 2’-fluoro modifications in place of the unstable 2’-OH of the ribose sugar, may reduce immunogenic effects and enhance both stability and activity of miR-34a [66]. However, chemical modifications should be carefully optimized to prevent interference with the silencing effect of miR-34a.

The first-in-human phase I clinical trial of MRX34, a liposomal miR-34a mimic, was initiated in 2013 (NCT01829971). Unfortunately, despite that MRX34 treatment was associated with acceptable safety and showed evidence of antitumor activity in a subset of patients [67,68], the trial was terminated by FDA in September of 2016 due to severe immune-related adverse events (AE). As reviewed [14], we believe that the MRX34’s AEs were caused by packaging vehicle-associated toxicity, too high doses of miR-34a, and unselected population of cancer patients recruited in the trial. The delivery vehicle of MRX34 was SMARTICLE, an amphoteric liposome composed of combinations of anionic and cationic lipids, which may induce systemic toxicity. Additionally, the MTD of MRX34 at 93–110 mg/m2 used in the clinical trial [67,68] corresponded to ~30 mg/kg body weight, which was 30X higher than preclinically effective doses (<1 mg/kg body weight) [65,69,70,71,72,73,74,75,76,77,78,79] (also see Table 1; below). Notably, the trial used MRX34 in an unselected mixed cohort of cancer patients instead of a specific subpopulation. Therefore, these three issues should be addressed in future miR-34a clinical translation.

### 5.2. Preclinical Studies of miR-34a in PCa

During the past decade, several translational studies of miR-34a, mainly using packaging vehicles for delivery, have focused on PCa (Table 1). Most of these preclinical studies used ≤2 mg/kg of miR-34a mimics (Table 1). The first preclinical study of miR-34a in PCa was back in 2011, when our group demonstrated that systemic delivery of miR-34a reduced tumor burden and lung metastasis by targeting CD44 in orthotopic PC-3 and LAPC9 xenografts [15]. In this study, miR-34a was complexed with a lipid-based delivery agent, MaxSuppressor In Vivo RNA-LANCER II. It is a formulation composed of neutral lipid, non-ionic detergent, and small molecules which enable highly efficient delivery of RNAi agents into animals. Additionally, the formulation has been shown to be well tolerable and does not induce an immune response [69]. This study [15] provided the proof-of-principle that miR-34a could be a promising therapeutic targeting PCSCs and PCa. A few years later, Gaur et al. utilized chitosan (CH) nanoparticles to deliver miR-34a via tail vein [47]. CH nanoparticles comprise biodegradable natural polysaccharides with low toxicity and immunogenicity [80,81]. In the study, systemic delivery of miR-34a robustly reduced the growth of prostate tumor in the bone. Importantly, comparing an intra-femoral PCa model to a sub-cutaneous model revealed that miR-34a delivery had more potent anti-tumor effects in the former, indicating that miR-34a may mediate tumor suppressive effects by targeting the bone microenvironment in addition to the tumor. [47]. The study highlights the clinical potential of miR-34a therapeutic for treating bone metastatic PCa.

The internalization mechanism of synthetic vehicle packaged with miR-34a involves the fusion of vehicle lipids with the cell membrane followed by endocytosis and subsequent release of miR-34a from the endosome into the cytosol. Additionally, cellular uptake of miR-34a is one of the important variables that impact anti-tumor efficacy. Wang et al. reported that ultrasound-induced microbubble cavitation (UIMC) improved anti-tumor efficacy by promoting the cellular uptake of miR-34a-loaded nanoparticles in PC-3 xenograft [82]. UIMC is a safe and effective technology widely used for drug and gene delivery [83]. Microbubbles exposed to ultrasound produce cavitation microfluidic field that enlarges the capillary gaps and cell membrane permeability, thus promoting the penetration of nanomedicine into tumor tissues [82]. In the study, biodegradable methoxy polyethylene glycol-polylacticco-glycolic acid-polylysine (mPEG-PLGA-PLL) nanoparticles was used to encapsulate miR-34a mimic. mPEG-PLGA-PLL nanoparticles is biocompatible, and the PLL in the structure can be used to adsorb miR-34a oligos. In addition, the mPEG layer on the surface of nanoparticles can increase the water solubility and prevents phagocytosis by the reticuloendothelial system in vivo [82]. Notably, biodistribution study showed that UIMC increased the accumulation of miR-34a-loaded nanoparticles in tumor tissues. Additionally, the delivery vehicle did not display obvious systemic toxicity with no significant weight loss and elevated biochemical parameters [82].

In addition, the concept of combinatorial therapy has been applied to miR-34a preclinical studies. For example, co-delivery of DOX and miR-34a via an amphiphilic micellar system achieved synergistic anti-tumor effect in DU145 xenograft model compared with DOX or miR-34a alone [84]. The delivery platform is a reducible self-assembling disulfide cross-linked stearyl-peptide-based micellar system (SHRss) using poly(l-arginine)-poly(l-histidine)-stearoyl as the copolymer building unit. The nanoscale SHRss micelles facilitated the escape of miR-34a from the endosome and release of DOX into the cell nucleus, and led to downregulation of SIRT1 and inhibition of DU145 and PC-3 cell proliferation [84]. DOX and miR-34a delivered by SHRss micelles mainly accumulated in the tumor tissue with some accumulation in the liver and spleen [84].

Interestingly, rubone, a chalcone derivative, was reported to be a miR-34a inducer to upregulate the intracellular miR-34a and elicit anti-cancer effects [61]. Co-delivery of rubone and paclitaxel via a micellar system significantly reduced the tumor burden in orthotopic paclitaxel-resistant PC-3 xenograft [61]. Mechanistically, rubone inhibited invasion and decreased the CSC population of paclitaxel-resistant PCa cells, resulting in the reversal of drug resistance. Additionally, rubone synergized with paclitaxel to promote drug effect by downregulating the levels of E-cadherin, SIRT1, as well as ALDH (aldehyde dehydrogenase) activity, which is a CSC marker [61]. Similarly, another study revealed that co-delivery of docetaxel and rubone by dual responsive micelles sensitized taxane and inhibited tumor growth in orthotopic taxane-resistant PC-3 xenograft [85]. This dual response micelle is a nano-platform responsive to pH and glutathione (GSH) levels that are altered in tumor microenvironment. Upon endocytosis by tumor cells, low pH value of endocytic vehicles promotes the dissociation of the micelles, leading to efficient drug release and sufficient exposure to GSH for the cleavage of disulfide bond [85]. The treatment with rubone micelles of taxane-resistant PCa cells robustly reduced the ALDH^hi^ CSC subpopulation in a dose dependent manner [85], indicating that miR-34a can reverse the chemoresistance by targeting PCSCs.

In a very recent study, recombinant adeno-associated virus (rAAV) was used as the delivery platform of miR-34a to investigate its therapeutic effect in transgenic adenocarcinoma mouse prostate (TRAMP) model [86]. rAAV-based gene therapy shows advantages of long-term expression, low immunogenicity, and nonchromosomal integration. Notably, a rAAV-based drug for the treatment of Leber’s congenital amaurosis was approved by the FDA in 2017 [87], suggesting potential clinical application of rAAV-based drug delivery in cancer. miR-34a expression was significantly downregulated in the anterior prostate (AP) and dorsal lateral prostate (DLP) of TRAMP mice compared to wild type (WT) mice [86]. Moreover, miR-34a expression is significantly decreased in mouse prostatic intraepithelial neoplasia (PIN) and prostate tumors compared to normal prostatic tissues, indicating a negative role of miR-34a in PCa development. After a single dose of intraprostatic injection, rAAV9-miR-34a improved the survival of TRAMP mice compared to mice that received PBS (median survival 307.5 and 220 days, respectively) [86]. miR-34a overexpression significantly reduced the neoplastic area in both AP and DLP, and downregulated cyclin D1 expression in TRAMP tumors [86]. These findings suggest that intraprostatic delivery of miR-34a effectively inhibits PCa progression *in vivo* by targeting cyclin D1. However, the body weight of rAAV9-miR-34a-treated group was markedly reduced after 30 weeks of injection [86], raising the safety concern of rAAV-based delivery platform.

**Table 1:** Preclinical studies of miR-34a in PCa.

Delivery System	Mouse Model	Route of Administration	Dose	Dose Schedule	Reference
Lipid-based transfection reagent	Orthotopic PC3 and DU145 xenografts	i.v.	1 mg/kg	Every 2 days for 5 times	[15]
Chitosan nanoparticle	s.c PC3MM2 xenograft; intra-femoral model	i.v.	250 μg/kg	Every 3 days for three weeks	[47]
Cationic polypeptide-based micelles	s.c DU145 xenograft	i.v.	2 mg/kg	Every 4 days for 4 times	[84]
PEG-PCD micelles	Orthotopic PC3-TXR xenograft	i.v.	10 mg/kg (Rubone)	Every 2 days for 5 times	[61]
pH and GSH responsive micelles	Orthotopic PC3-TXR xenograft	i.v.	25 mg/kg (Rubone)	Every 2 days for 9 times	[85]
mPEG-PLGA-PLL nanoparticles	s.c. PC3 xenograft	i.v.	1.5 mg/kg	Every 3 days for 7 times	[82]
rAAV9-miR-34a vector	TRAMP genetic model	i.p.	1 × 10^11^ genome copy	Single dose	[86]

s.c.: subcutaneous injection; i.v.: intravenous injection; i.p.: intraprostatic injection.

### 5.3. Novel miR-34a Delivery Systems for Targeting PCa

One of the apparent limitations using packaged vehicles is the lack of specific delivery of miR-34a to tumors. This could be the cause of systemic toxicity due to off-target effects. Several targeted delivery approaches have been developed to overcome the challenge. One strategy is via ligand-mediated delivery of miRNA mimics. The principle is direct conjugation of a targeting ligand to the miRNA in the absence of a delivery vehicle. When designing a ligand-conjugated miRNA therapeutic, several critical features should be considered (reviewed in [66]). To achieve specificity, the ligand of choice should bind to a high-affinity receptor that is highly expressed on tumor cell surface. Moreover, the same receptor should not be expressed, or expressed at a relatively low level, on normal cells, or should be inconsequential for targeted delivery. In order to establish the potent interaction between the ligand and the receptor, it should also be taken into consideration to include a linker between the ligand and the miRNA. The linker itself can be chemically modified to improve binding affinity and the pharmacokinetics properties of the ligand. Chemical modification of the miRNA can also be considered to improve serum stability and increase intracellular half-life. Several small molecule ligands have been developed for delivery of miR-34a specifically and robustly to tumor cells. One good example is folate, an essential vitamin and a high-affinity ligand for the folate receptor (FR) [88]. FR expression is highly upregulated in several cancer types including ovarian, lung and breast cancers [66]. Orellana et al. were the first to directly conjugate miR-34a to folate (folate-miR-34a), and they showed that folate-miR-34a is selectively targeted to FR-expressing tumors, downregulates target genes, and suppressed the growth of lung and breast cancer models *in vivo* [65].

Prostate-specific membrane antigen (PSMA) is a promising therapeutic target for advanced PCa. PSMA, also known as folate hydrolase 1 (FOLH1), is a glycoprotein originally discovered on the membrane of prostatic epithelial cells [89]. PSMA is highly, but heterogeneously, upregulated in PCa cells [89,90]. It is also expressed in the neovasculature of several solid tumors and its expression has been associated with PCa progression [90,91,92]. Currently two PSMA-targeted Positron Emission Tomography (PET) imaging drugs, piflufolastat F-18 and Ga-68 PSMA-11, have been approved by FDA for men with suspected PCa metastasis. As radioactive drugs that emit positrons, PET imaging using these probes can indicate the presence of PSMA-positive PCa metastases in the patient’s body. Furthermore, multiple PSMA-targeting therapies are currently in clinical development, including radioligand therapy, PSMA-targeting immunotherapies (bi- and tri-specific T-cell engagers), antibody-drug conjugates, photodynamic therapy, imaging-guided surgery, and ultrasound-mediated nanobubble destruction (reviewed in [92,93]). The study led by Liu et al. demonstrated that PSMA is constitutively internalized via endocytosis and that the rate of internalization is increased by binding to anti-PSMA antibodies [66]. Following internalization, PSMA undergoes receptor cycling which allows additional internalization cycles [66]. With the consideration of the key features of ligand-conjugated miRNA therapeutics, PSMA may represent a golden receptor for specific targeting of PCa.

Interestingly, DUPA, a synthetic urea-based ligand, can bind to PSMA with nanomolar affinity, saturating the receptor quickly [90], suggesting DUPA could be an ideal ligand for conjugated miR-34a therapeutic to target PCa. In fact, DUPA was used to specifically deliver siRNAs to PSMA-expressing PCa cells both *in vitro* and *in vivo* [94]. However, therapeutic efficacy of DUPA-siRNA conjugates needs to be further studied. Notably, PSMA expression is increased in advanced-stage PCa and CRPC [95]. Moreover, treatment with Enza increased PSMA expression of PSMA-low PCa [96]. Therefore, DUPA-conjugated miR-34a could be a novel PSMA-targeted therapeutic for aggressive PCa by targeting treatment-reprogramed and PSMA-expressing PCSCs.

## 6. Conclusions and Perspectives

miR-34a is a bona fide tumor suppressor that represses PCa development by cooperating with p53 in genetic mouse models. In addition, miR-34a, commonly underexpressed in PCSCs, is a potent PCSC inhibitor by targeting cellular processes essential for CSC survival and functions including invasiveness and metastasis, stemness, epigenome, and cell survival. These discussions suggest that miR-34a could be developed into a specific anti-PCSC therapeutic for treating aggressive (advanced, metastatic, and therapy-resistant) PCa that are highly enriched in PCSCs. In reality, optimal miR-34a delivery specifically to prostate tumors still represents a major challenge for its clinical translation. Great efforts have been made towards the use of packaging vehicles for miR-34a delivery for PCa treatment. However, vehicle-associated toxicity, reduced stability, and lack of specificity are the main roadblocks that limit the clinical development of packaged miR-34a therapeutics. In this regard, ligand-mediated delivery of miR-34a represents a promising strategy to achieve specific targeting to prostate tumors while minimizing systemic toxicity. PSMA is highly expressed in PCa, and continuously expressed and upregulated during progression and therapeutic treatment, which makes it an attractive target in PCa. Therefore, DUPA-conjugated miR-34a could be a potential anti-CSC therapeutic for aggressive PCa that continue to express PSMA. In-depth preclinical studies are required to validate the efficacy and safety of this approach prior to its clinical application. In addition, chemical modification of the miR-34a mimics represents another future avenue to improve its serum stability.

## List of Abbreviations

PCa	Prostate Cancer
AR	Androgen Receptor
CRPC	Castration Resistant Prostate Cancer
CSCs	Cancer Stem Cells
PCSCs	Prostate Cancer Stem Cells
miR-34a	microRNA-34a
AUA	American Urological Association
GS	Gleason Score
LN	Lymph Node
ARSIs	AR Signaling Inhibitors
ADT	Androgen-Deprivation Therapy
Enza	Enzalutamide
metastatic CRPC	mCRPC
miRNAs	microRNAs
nt	Nucleotide
pri-miRNA	primary miRNA
mRNAs	Messenger RNAs
3’-UTR	3’-Untranslated Regions
TCGA	The Cancer Genome Atlas
T	Tumor
RISC	RNA-Induced Silencing Complex
lncRNAs	Long non-coding RNAs
DOX	Doxorubicin
PRC2	Polycomb Repressive Complex 2
5Aza-2′dC	5-aza-2′deoxycytidine
EMT	Epithelial-to-Mesenchymal Transition
STMN1	Stathmin 1
TCF	T-Cell Factor
LEF	Lymphoid Enhancer Factor
SIRT1	Sirtuin-1
HuR	Human antigen R protein
AE	Adverse Events
UIMC	Ultrasound Induces Microbubble Cavitation
mPEG-PLGA-PLL	Methoxy polyethylene glycol-polylacticco-glycolic acid-polylysine
SHRss	Self-assembling disulfide cross-linked stearyl-peptide-based micellar system
GSH	Glutathione
rAAV	Recombinant Adeno-Associated Virus
TRAMP	Transgenic Adenocarcinoma Mouse Prostate
AP	Anterior Prostate
DLP	Dorsal Lateral Prostate
WT	Wild Type
PIN	Prostatic Intraepithelial Neoplasia
FR	Folate Receptor
PSMA	Prostate-Specific Membrane Antigen
FOLH1	Folate Hydrolase 1
PET	Positron Emission Tomography
